# Tests Demonstrate the Accuracy of the *Q*_*im*_-Reaction Path Hamiltonian for Double-Well Tunneling Splittings for H‑Atom Transfer

**DOI:** 10.1021/acs.jpca.6c01085

**Published:** 2026-04-03

**Authors:** Chen Qu, Apurba Nandi, Paul L. Houston, Joel M. Bowman

**Affiliations:** † Independent Researcher, Toronto, Ontario M9B0E3, Canada; ‡ Department of Chemistry and Cherry L. Emerson Center for Scientific Computation, 1371 Emory University, Atlanta, Georgia 30322, United States; § Department of Chemistry and Chemical Biology, 138309 Cornell University, Ithaca, New York 14853, United States

## Abstract

Reduced dimensional models of tunneling have been a focus of theoretical chemistry since the birth of this field. The simplest model is the “reaction path” that connects a saddle point to the associated minima describing the reactant and the product. The widely used intrinsic reaction coordinate is a curved path that, perforce, contains a complex kinetic energy operator. In this paper, we examine a rectilinear reaction path, denoted the *Q*_*im*_-path, and the associated 1d Hamiltonian. In the simplest version, the generally small Coriolis coupling terms are neglected. This Hamiltonian was introduced and shown to produce tunneling splittings of H- and D-transfer of malonaldehyde with good accuracy by Wang and Bowman in 2008. Here, we present further tests of this approach for tunneling splittings of H- and D-transfer for a number of molecules with symmetric double wells. These are tropolone, acetylacetone, malonaldehyde, and protonated oxalate anion, for which high-quality machine-learned potentials have been reported. Tests make use of the available benchmark results, including those from diffusion Monte Carlo calculations and, for tropolone, ring polymer instanton results. Extensions of the method for asymmetric double wells due to asymmetric isotope substitution and potential asymmetry, as well as to more than one degree of freedom, are reported.

## Graphical abstract

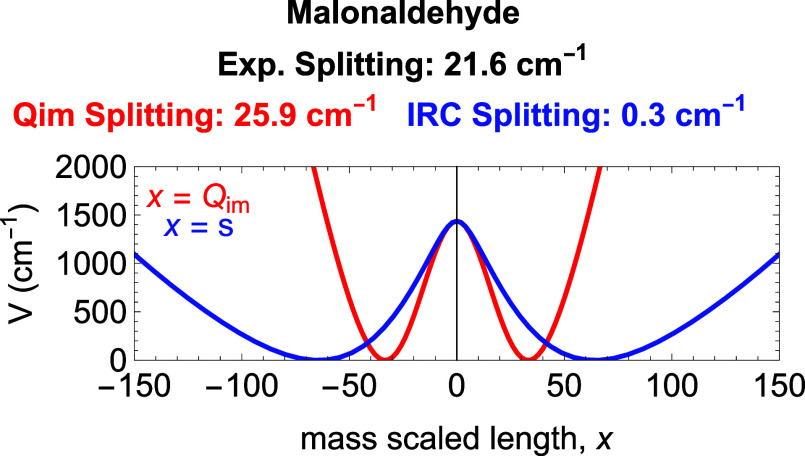

## Introduction

The reaction path, which connects a saddle point to the associated minima describing the reactant and the product, is a central concept in chemistry. The best known reaction path is the Intrinsic Reaction Coordinate (IRC) of Fukui,^1^ usually denoted *s*, and the associated Reaction Path Hamiltonian. That Hamiltonian, derived originally for collinear (2 degree-of-freedom) A+BC reactions in 1966 ^2^ contains the potential along the path, *V*(*s*), plus the kinetic energy operator, which has curvature terms. Given the complexity (at the time) of this 2d-Hamiltonian, two approximations were introduced. One is the vibrationally adiabatic (VA) approximation, in which motion orthogonal to the path is approximated by a sum of separable, typically harmonic oscillator Hamiltonians, and a zero-curvature or approximate curvature treatment of the path. Curvature terms in the kinetic energy operator cause nonadiabatic vibrational coupling that violate the assumption of the VA approximation. These two approximations finally result in a 1d Hamiltonian given by a simple kinetic energy operator plus an effective potential, given by the sum of the potential along *s*, *V*(*s*), plus the local adiabatic vibrational energy. Many tests of this approximate, simple effective 1d Hamiltonian have been made, with the first rigorous one for collinear H+H_2_ in 1972.^3^ Numerous extensions of the IRC to account for curvature and corner cutting have been made over the years.^4^

The general reaction path Hamiltonian for polyatomic molecules was derived in 1979.^5^ and the nonadiabatic terms in the Hamiltonian were derived in generality. The zero-curvature and the VA approximations, made in that paper, have become widespread in the field as the general treatment of the vibrational adiabaticity for chemical reactions. Specifically, for the case of the zero-point motion, the approximations result in an effective 1d Hamiltonian in *s*. For the simplest case, the path-dependent total zero-point energy (ZPE) is added to *V*(*s*) to obtain an 1d effective potential. The total ZPE is just the sum of the ZPEs of each mode orthogonal to the path. These modes are typically treated in the harmonic approximation. For polyatomic molecules such as the ones considered in this paper, i.e., with nine and more atoms, there is considerable computational overhead to evaluate the path dependent separable normal modes, even within the zero-curvature approximation. In addition, another vexing aspect of this approximation is that while some modes may be adiabatic others are not; see ref 6 for a detailed discussion of this for malonaldehyde.

Given the above complications of the approximations made to the reaction path Hamiltonian, Wang and Bowman in 2008 introduced a much simpler reaction path and 1d-Hamiltonian. The path is a rectilinear one (and thus with no “curvature terms”) along the direction of the imaginary-frequency normal-mode coordinate of the saddle point; this path variable is denoted by *Q*_*im*_.^7^ The corresponding 1d Hamiltonian in this variable is very simple, provided the vibrational angular momentum terms in the rigorous Watson Hamiltonian^8^ are ignored. This is a widespread and generally accurate approximation in the more conventional uses of this Hamiltonian for vibrational dynamics of molecules, using the normal modes of a minimum.^9,10^ Note that the approximate vibrationally adiabatic treatment of modes orthogonal to the path is not used. In the 2008 paper tunneling splittings for malonaldehyde were calculated and compared to benchmark calculations, using this simple Hamiltonian and also the one included ZPE along the path. The results, which are reviewed and discussed below, indicated that the addition of the local ZPE led to less accurate results for H-atom transfer.

Further tests and extensions of the simple 1d Hamiltonian for splittings are the focus of this paper and are made for a variety of molecules, including two that are larger than malonaldehyde. Extensions of the method are made for asymmetric double wells and inclusion of an additional degree of freedom.

The paper is organized as follows. In the next section, we give and discuss the *Q*_*im*_ 1d Hamiltonian starting from the rigorous Watson Hamiltonian. We then present general computational details for implementation of the method with a focus on tunneling splittings. This is followed by results for a number of symmetric double well molecules, where tests of the 1d *Q*_*im*_ are given against benchmark results. An illustration to a 2d approach is given for protonated oxalate anion. Asymmetric double wells, owing to electronic asymmetry and asymmetric isotopic substitution are then considered. A general discussion of the *Q*_*im*_-path method is provided, including remarks about the ease of using the method with limited ab initio calculations. A brief summary and conclusions complete the paper.

## Methods

### *Q*_*im*_-Path Hamiltonian

To begin consider the exact Watson Hamiltonian,^8^ given by

$$\hat{H}=\frac{1}{2}\sum_{\alpha \beta }({\hat{J}}_{\alpha }-{\hat{\pi }}_{\alpha })\mu_{\alpha \beta }({\hat{J}}_{\beta }-{\hat{\pi }}_{\beta })-\frac{1}{2}\sum_{k}^{F}\frac{\partial^{2}}{\partial Q_{k}^{2}}+V(Q)-\frac{1}{8}\sum_{\alpha }\mu_{\alpha \alpha }$$ 1

where α­(β) represent the *x*, *y*, *z* coordinates, ${\hat{J}}_{\alpha }$ and ${\hat{\pi }}_{\alpha }$ are the components of the total and vibrational angular momenta respectively, μ_αβ_ is the inverse of effective moment of inertia, and *V*(***Q***) is the potential, which depends on the *F* mass-scaled normal-mode coordinates ***Q*** which, for nonlinear molecules, equals 3*N* – 6. The last term, the “Watson term”, is typically ignored, as it is generally very small and essentially a constant, equal to its value at the reference configuration.

This Hamiltonian, while generally used for the vibrational modes of a minimum, can and has been used in all the modes at a saddle point separating two wells.^11,12^ The 1d approximation to this Hamiltonian uses just the imaginary-frequency normal mode as the “reaction coordinate”. The 1d-Hamiltonian is given by

$$\hat{H}=\frac{1}{2}\sum_{\alpha \beta }{\hat{\pi }}_{\alpha }\mu_{\alpha \beta }{\hat{\pi }}_{\beta }-\frac{1}{2}\frac{\partial^{2}}{\partial Q_{im}^{2}}+V(Q_{im})$$ 2

The first term contains the so-called vibrational angular momentum (VAM) terms that depend on the normal coordinates and momenta and the inverse of the effective moment of inertia. In many instances, i.e., molecules with small rotation constants, these terms are small and so are ignored. We discuss this approximation in more detail later. *V*(*Q*_*im*_) is the potential relaxed with respect to the remaining 3*N* – 7 saddle-point normal coordinates at each value of *Q*_*im*_. Thus, the working 1d Schrödinger equation is

$$\left[-\frac{1}{2}\frac{\partial^{2}}{\partial Q_{im}^{2}}+V(Q_{im})\right]\psi_{n}(Q_{im})=E_{n}\psi_{n}(Q_{im})$$ 3

The methods to obtain the relaxed *V*(*Q_im_*) over a range spanning the saddle point, minima and repulsive region are given in the next section, along with the method to obtain the energies and wave functions. Note that this simple Hamiltonian does not employ the approximate vibrational adiabaticity assumption. And thus, zero-point energy in modes orthogonal to the path is not included. We return to this point below, where this adiabatic treatment was tested for.

Before moving to that section, it is of interest to compare the relaxed potentials *V*(*s*) and *V*(*Q*_*im*_) for malonaldehyde. The upper panel of Figure 1 plots of *V*(*Q*_*im*_) and *V*(*s*) for malonaldehyde. The major point to make about these plots is the large difference in their shapes. Both begin at the H-atom transfer saddle point at their respective zero values, and both reach the symmetric minima at very different values. The curvilinear path length *s* is obtained in the usual way as the length of path in the full 3*N* – 6 dimensional space, and so in general *s* is larger than *Q_im_*. And it is worth noting that the so-called zero-curvature 1d Hamiltonian (with no consideration of modes orthogonal to it) is given by eq 3 and replacing *V*(*Q*_*im*_) with *V*(*s*). Clearly, doing this will (and does) result in a smaller ground state tunneling splitting than the one using *V*(*Q*_*im*_) as shown in ref 7. The numbers are 25.9 and 4.6 cm^–1^ for H and D transfer, respectively using *V*(*Q*_*im*_), and the corresponding ones using *V*(*s*) are 0.3 and 0.1 cm^–1^. These calculations were done with an a accurate CCSD­(T)/CBS-based potential energy surface,^13^ and so the good agreement with experimental splittings of 21.6 and 2.9 cm^–1^ for H and D, respectively, for the *V*(*Q_im_*) is not fortuitous. The lower panel of Figure 1 indicates the molecular motion along the relaxed *Q*_*im*_-path.

**Figure 1:**
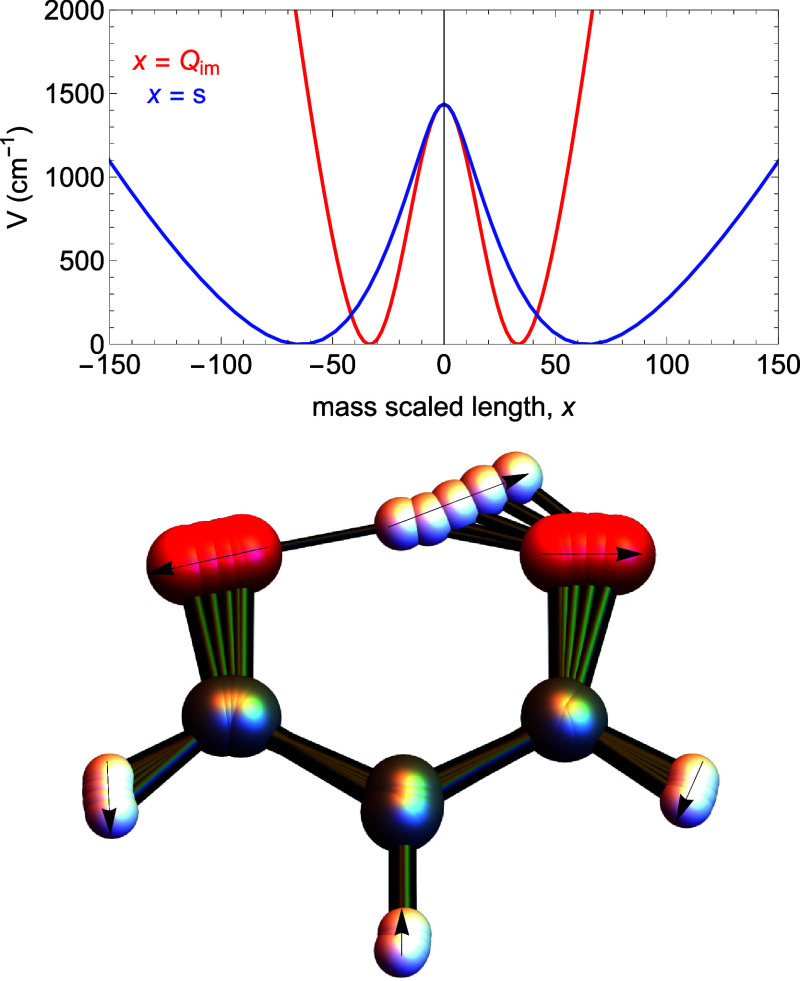
(Upper) The 1d potential curve for H-transfer in malonaldehyde as a function of mass-scaled length *Q*_*im*_ (red) and *s* (blue). (Lower) The motion of the malonaldehyde along the relaxed path. The arrows show the direction of motion for five geometries of malonaldehyde as *Q*_*im*_ goes from 0 to 72 and *s* goes from 0 to 214. In both cases, the potential starts at the TS with 1424 cm^–1^. It is 584 cm^–1^ at step 2, 18 cm^–1^ at step 3, 925 cm^–1^ at step 4, and 2509 cm^–1^ at step 5.

Based on these early results, we have used *V*(*Q*_*im*_) for a number of applications of tunneling splittings and the results and new tests are given below.

We conclude this section with comments on extending the 1d approach to more degrees of freedom and also inclusion of vibrational angular momentum terms. First, consider the extension to more degrees of freedom.^14^ In this approach the full potential is relaxed holding *M* coordinates, always including *Q*_*im*_, fixed. The Hamiltonian in this case is give by

$$\hat{H}=-\frac{1}{2}\sum_{i}^{M}\frac{\partial^{2}}{\partial Q_{i}^{2}}+V(Q_{im},\cdot \cdot \cdot ,Q_{M})$$ 4

*V*(*Q*_*im*_, ···, *Q*_*M*_) is the relaxed potential with respect to the remaining modes. Here we apply this for one additional normal coordinate held fixed for protonated oxalate anion.

The importance of the VAM terms was demonstrated in full-dimensional variational calculations of tunneling splittings in H_3_O^+^ using saddle point normal modes.^12^ The double-well motion in this case is large amplitude “umbrella” motion connecting the two *C*_3*v*_ minima. Karmarchik et al. showed how these terms can be included in the reduced dimensionality Hamiltonian, eq 4, to obtain accurate splittings.^14^ That these terms are important for H_3_O^+^ is not surprising, given the relatively large rotation constants and the large amplitude motion of three H atoms. However, for H-atom transfer reactions in larger molecules, which are the focus of this paper, these terms can be safely ignored, as demonstrated already for malonaldehyde in diffusion Monte Carlo^13^ and MCTDH calculations.^15^

### Computational Details

The first step in developing *Q*_*im*_ as a reaction coordinate is to determine the geometry and energy of the transition state (TS) and to perform a normal-mode analysis (NMA) at this geometry. The eigenvalues of the Hessian using mass-weighted coordinates in atomic units typically produce 6 zero-frequency modes: one imaginary frequency mode, and 3*N* – 7 real frequency modes, where *N* is the number of atoms. The eigenvectors provide unit vectors in the directions of the normal modes. We focus on the imaginary frequency and its corresponding unit vector. Let this vector be denoted by ${\vec{q}}_{im}$ and then *Q*_*im*_ is the scaler distance along this vector. This is the 1d reaction coordinate. To get the relaxed potential *V*(*Q*_*im*_) at any value of *Q*_*im*_ the total potential is minimized with respect to the remaining 3*N* – 7 TS normal modes. We used Newton’s method for this constrained minimization. To obtain the full 1d *V*(*Q*_*im*_), we start at *Q*_*im*_ = 0 (TS), and perform the constrained optimization at a series of *Q*_*im*_ values with an increment of 0.05 atomic units. This leads to roughly 2000 optimizations per molecule (only the positive or negative half of the potential is needed because it is symmetric), and can be done within a few minutes. Then spline interpolation is used in DVR (discrete variable representation) computations of the energies and wave functions. We note that our choice of the *Q*_*im*_ grids is perhaps unnecessarily dense (we used such a large number simply because the computations are so “cheap”); a few hundred points are more than enough to ensure a good spline fit. Also, note that *V*(*Q*_*im*_) must be obtained into the repulsive region beyond the minimum in order to be used in the 1d Schrödinger equation, eq 3, as already noted. For the 2-d reaction surface, we perform this minimization for fixed values of *Q*_*im*_ and one TS normal mode, which we describe in detail in the next section.

It is worth noting that the portion of *V*(*Q*_*im*_) from the saddle-point to the minimum can be obtained from the standard IRC, which is available in many electronic structure codes. Since the IRC and TS normal modes are both expressed in mass-scaled Cartesian coordinates it is straightforward to get one from the other. We return to this point in more detail below, where we propose and test a suggestion to augment the information from the IRC to obtain *V*(*Q*_*im*_) beyond the minimum.

The solutions to the Schrödinger equation, eq 3, are obtained using the efficient DVR.^16,17^ For this method to give accurate results, energies and geometries for values of *Q*_*im*_ beyond those of the global minimum are required, otherwise there are no outer walls to define the potential. These energies and geometries are obtained using the same minimization method used from the saddle point to the minima.

All of our calculations make use of full-dimensional PESs. However, as noted above the portion of the *Q*_*im*_-path and corresponding *V*(*Q*_*im*_) are directly available from the IRC and *V*(*s*). A method to extend these data to the repulsive wall without the need of a full PES would obviously be very useful. We propose and test a method using a separable harmonic potential at the minimum in terms of the corresponding normal modes and frequencies. This information is readily available from electronic structure codes. This harmonic potential can be used to obtain the relaxed repulsive potential beyond the minimum. Of course this is an approximation to the true potential; however, it should be adequate for low lying eigenstates and eigenvalues of the 1d *Q*_*im*_ Hamiltonian. Results using this approximate method are compared to the *Q*_*im*_ results using a full PES in the following section.

With *V*(*Q*_*im*_) defined, determination of the energy levels and wave functions by the DVR method is straightforward. Because *Q*_*im*_ is mass-weighted in atomic units, *ℏ* and the mass are unity and one has only to establish a grid, enter the Hamiltonian elements and diagonalize. We note that it is also possible to use DVR for potentials in two or more dimensions.^17^ The disadvantages for multiple dimensions are (1) that the Hamiltonian matrix can get quite large, and (2) that it is often difficult to perform the relaxation for geometries that have high energies. The advantage is that one obtains tunneling splittings associated with the additional mode. An example will be provided below.

## Results

### Symmetric Double Wells

Figure 2 shows four examples of using the relaxed 1d potential *V*(*Q*_*im*_) in conjunction with the DVR method^16,17^ to determine the tunneling splittings via direct calculation of the energy eigenvalues. The structures at the transition states for these examples are shown in Figure 3. The splittings in the first pairs of levels in all panels are too small to resolve at the scale of the figures but are given as follows. Panel (a) shows the lower six energy levels and the *Q*_*im*_-path for H transfer in tropolone, where the barrier is 2525 cm^–1^.^18^ The magnitude of the splitting at the two lowest levels is about 2 cm^–1^, whereas experimental values are about 1 cm^–1^.^19,20^ In panel (b) the similar information for H transfer in acetylacetone is shown, where the barrier is 1119 cm^–1^ and the splitting between the lowest two levels is about 38 cm^–1^. In this case the two methyl rotors have been fixed at the saddle point values. Panel (c) shows the *Q*_*im*_ path for H transfer in malonaldehyde^13^ where the splitting between the two lowest two levels is about 26 cm^–1^ and the experimental value is 22 cm^–1^.^21,22^ Panel (d) shows the double well for H transfer in the protonated oxalate, where the splitting of the lowest two levels is about 33 cm^–1^. The potential for protonated oxalate has recently been reported.^23^ Figure 2 shows *V*(*Q*_*im*_) and the a barrier is 1172 cm^–1^. A second calculation, based on CCSD­(T)/CBS calculations at geometries corresponding to the original *Q*_*im*_ path, gave a somewhat higher barrier of 1250 cm^–1^ and a splitting of 28 cm^–1^.

**Figure 2:**
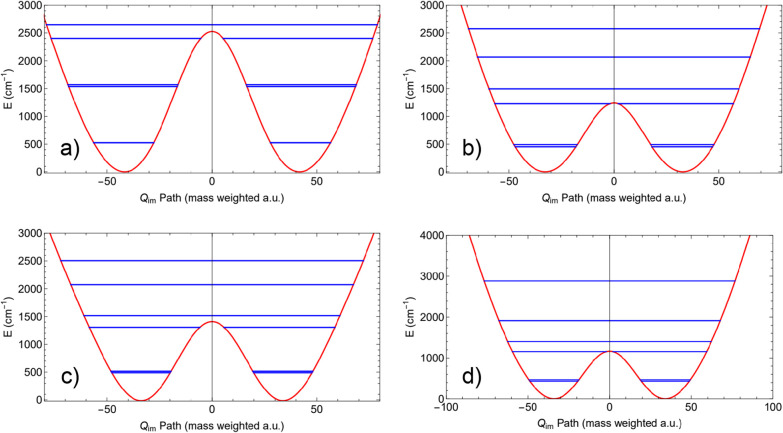
Four examples of the 1d potential *V*(*Q*_*im*_) for calculating tunneling splittings. (a) tropolone, (b) acetylacetone, (c) malonaldehyde, and (d) protonated oxalate. See text or Table 1 for the lowest splittings, which are too small to resolve in this figure.

**Figure 3:**
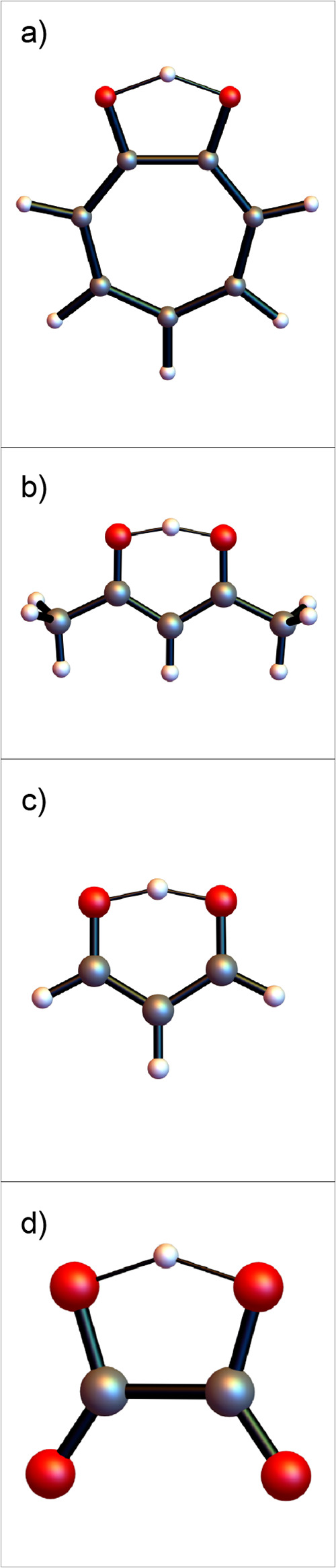
Transition state structures for molecules with double wells: (a) tropolone, (b) acetylacetone, (c) malonaldehyde, and (d) protonated oxalate (carbon = gray, oxygen = red, hydrogen = white).

The comparisons with available experimental values are good, but it is more instructive to compare the results of the *Q*_*im*_-method to other theoretical methods using the same or similar potential energy surfaces. Three methods are commonly used. The DMC method is normally used to determine the ZPE, but with the fixed-node approximation it can also determine the energy of the first excited state. This approach is probably the most accurate one *provided* the splitting is larger than the statistical uncertainty, which is typically several wavenumbers. For smaller splittings, and especially for very small ones, e.g., 0.014 cm^–1^ for the formic acid dimer, the ring-polymer instanton (RPI) method^24–26^ is currently the method of choice; however, it is limited to the ground vibrational state splitting. Ideas to augment that method with vibration configurations interaction for excited state splitting are in development.^27^ Finally, high-level vibrational configuration interaction calculations (VSCF/VCI) of the spectrum with programs such as MULTIMODE (MM)^9,10^ can also accurately calculate splittings and for the ground vibrational state as well as excited states. These are challenging calculations in full dimensionality, and current applications have been made to ammonia and hydronium. Results from these methods for a variety of molecules and splittings provide tests of the *Q*_*im*_ method. These are given in Table 1 in order of decreasing molecular size. For some cases in the table, different PESs, with different barrier heights, were used. The table shows variations in the electronic barrier height from 690 to 2512 cm^–1^ and corresponding variations in ground state tunneling splitting from roughly 1 to more than 100 cm^–1^. This large splitting is seen for acetylacetone, which has the smallest barrier, 763 cm^–1^, based on MP2 energies. As discussed in detail previously,^28^ the splitting in this molecule is a major challenge for the *Q*_*im*_ method because the two methyl rotors have slightly different configurations at the saddle point and global minimum. This relaxation cannot be captured by the rectilinear *Q*_*im*_ path and so the barrier is not equal to the one experienced by the DMC method. There is a more recent set of *Q*_*im*_ and DMC splittings for acetylacetone using a Δ-ML PIP potential with a LCCSD­(T)-F12/cc-pVTZ-F12 barrier height of 1234 cm^–1^.^29^ Those results are given in Table 1 and details of those calculations are given in ref 29.

**Table 1:** Comparison of *Q*_im_ Tunneling Splittings (cm^–1^) with Benchmarks for H­(D)-Atom Tunneling for Indicated Molecules and Corresponding Double-Well Barriers

	barrier (cm^–1^)	tunneling splitting (cm^–1^)			
molecule		*Q_im_* method	benchmark	method	ref
tropolone					
(H)	2055	3.9	2.7	RPI	^30^
(H)	2512	2.3	0.92	RPI	^30^
(H,^16^O^18^O)	2512	4.3^a^	2.0	RPI	^30^
acetylacetone					
(H)	763	113	156–160	DMC	^28^
(H)	1234	38	32^b^	DMC	^29^
(D)	763	41	40–43	DMC	^28^
(D)	1234	8.2	–^c^	DMC	^29^
malonaldehyde					
(H)	1409	26	22	DMC	^13^
(D)	1409	4.6	2.6	DMC	^13^
protonated oxalate					
(H,1d)	1172	33	35	RPI	^31^
			36	DMC	^32^
(H,2d)	1172	33^e^			
(H,1d)	1250	28^a^			
(D,1d)	1172	6.6^e^	5.6^e^	DMC	
ammonia					
(H)	1820	0.18	0.60	VSCF/VCI	^14^
hydronium					
(H)	690	19/50^d^	46	VSCF/VCI	^14^

a Current work based on a CCSD­(T)/CBS barrier height.^32^

b Estimated, see text for details.

c The *Q*_*im*_ result is smaller than the uncertainty in the DMC calculations.

d Includes vibrational angular momentum terms.

e Current work.

The *Q*_*im*_ results given for ammonia and hydronium stand apart from the others as the motion is not an H-atom transfer, but a large amplitude umbrella motion involving the three H atoms. As seen, the splitting for ammonia is less than 1 cm^–1^, whereas the one for hydronium is much larger. Also, note that hydronium is the one example where the vibrational angular momentum terms were added to the 1d *Q*_*im*_-Hamiltonian.^14^ Including these increases the accuracy of the splitting considerably compared to the benchmark one.

In summary, excluding results for ammonia and hydronium, which are not H-atom transfer cases, the range of benchmark splittings is 0.92 to roughly 158 cm^–1^. The corresponding range of *Q*_*im*_ splittings is 2.3–113 cm^–1^. Figure 4 provides a visualization of the data in Table 1. As seen, the 1d-*Q*_*im*_ splittings track the benchmark ones well.

**Figure 4:**
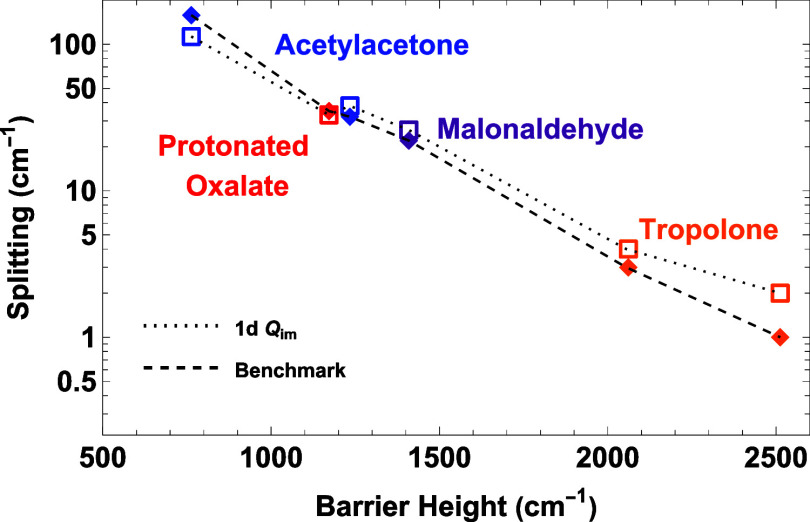
Semilog plot of benchmark and 1d *Q*_*im*_ splittings versus indicated barrier height for potentials of indicated molecules. See text for more details.

Next we present a test using the harmonic potential of the minimum to extend the *Q*_*im*_ path from the symmetric minimum to the repulsive region of *V*(*Q*_*im*_) for protonated oxalate. This is shown in Figure 5, where, as expected, the harmonic potential deviates increasingly from the accurate potential for larger displacements from the minima. Tunneling splittings, in cm^–1^, were obtained for both potentials from the 1d DVR eigenvalues, with the following results for the first two pair of splittings (value using PES as opposed to harmonic approximation in parentheses): 34.7(33.1), 264.6(241.3). The accuracy for the most relevant ground state is within a few percent of the accurate result. As expected, deviations increase for the excited state splittings.

**Figure 5:**
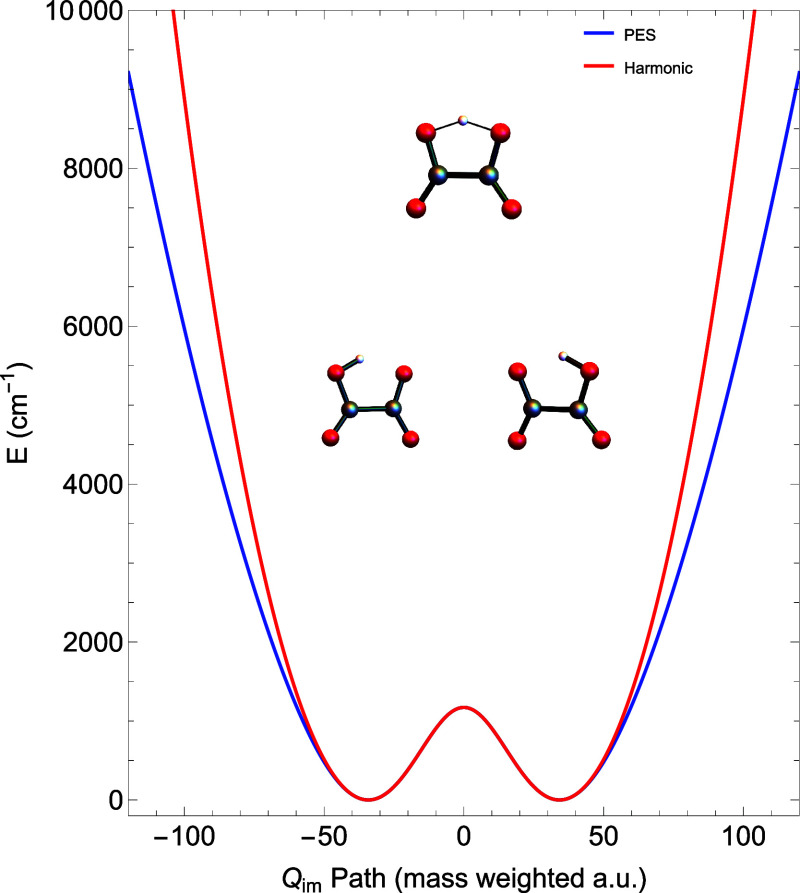
*V*(*Q*_*im*_) for protonated oxalate from PIP MLP (PES)^23^ and the harmonic oscillator (Harmonic) potential for the repulsive regions beyond the minima. See text for details.

The protonated oxalate also provides an example of using a 2d projection of the minimum energy path. We did this in order to learn how tunneling causes splittings in levels of other modes and also to investigate “corner-cutting”, as this can occur in 2d. We use *Q*_*im*_ and *Q*_1_ as the two coordinates and perform an optimization of the remaining 3*N* – 8 normal coordinates at each value of *Q*_*im*_ between −100. and 100. and at each value of *Q*_1_ between −72 and 40. For this discussion, the *Q*_1_ mode of the TS is motion along the *C*_2_ symmetry axis and has the highest frequency of the TS modes.

The potential surface is shown in colored contour form in Figure 6. The grid spacing for the diagram is 0.5 in each dimension. A 2d DVR calculation was performed for grid spacings of 2.5 and 1.0 with little difference in the values of the lower vibrational levels, indicating that even the coarser grid provides converged results. The figure also shows in black contours the wave function amplitude corresponding to the lowest vibrational level. The splitting between the two lowest vibrational levels is 33.2 cm^–1^, in excellent agreement with the 1D calculation. Even though the lowest energy level, at about 319 cm^–1^, is significantly lower than the barrier of 1172 cm^–1^, there is substantial wave function amplitude at the position of the barrier, indicating that tunneling under it is fairly facile. In addition, we note that the wave function amplitude seems centered on the transition state, indicating that little “corner-cutting” takes place in the *Q*_1_ dimension. Negligible corner-cutting is likely because the potential increases strongly along *Q*_1_ from the transition state, in accord with the vibrational frequency of about 2100 cm^–1^ for this mode. The energies and assignments for the lowest 13 vibrational levels are provided in Table 2, where the assignments were made by examination of the wave functions. The splitting between the (*Q*_*im*_,*Q*_1_) = (0,1) and (1,1) levels is 69.5 cm^–1^.

**Figure 6:**
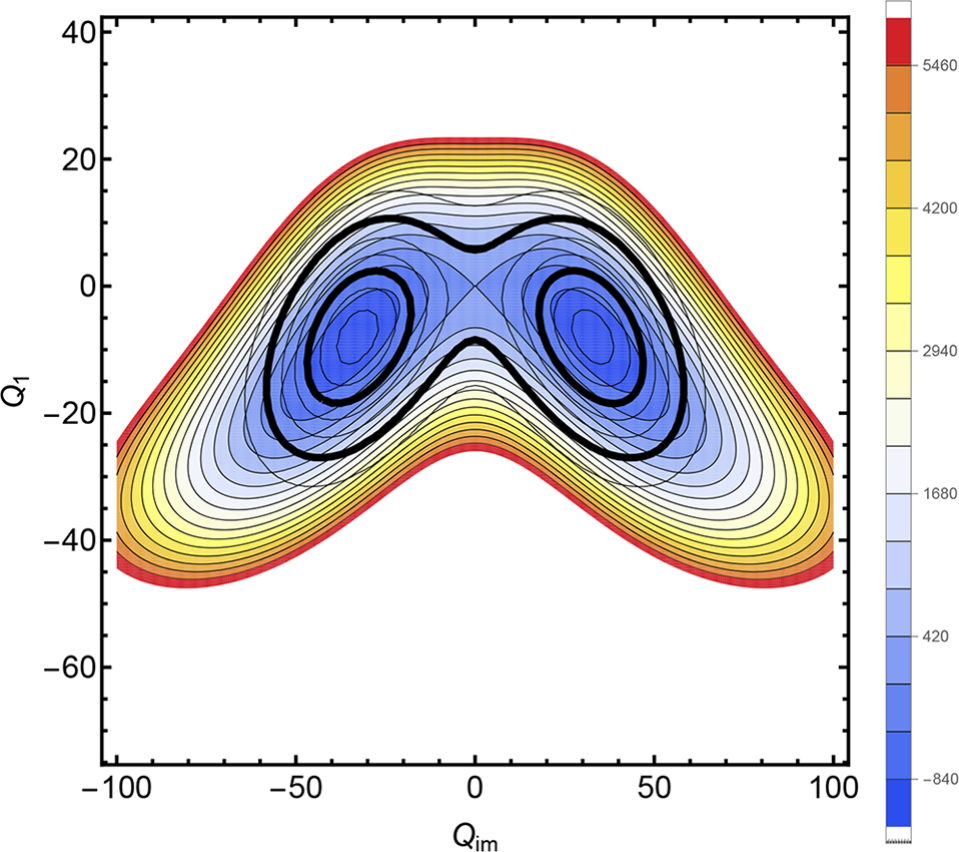
Two-dimensional contour plot of the potential Energy Surface for C_2_O_4_H^–^ as a function of *Q*_*im*_ and *Q*_1_ in color with all other normal modes optimized at each point. Contour plot of the wave function of the lowest vibrational level in black. The energy scale is in cm^–1^. The wave function contour amplitude spacing is 0.01.

**Table 2:** Lowest Vibrational Energy Levels for C_2_O_4_H^–^ in the Relaxed 2d Potential Shown in Figure 6

level	energy (cm^–1^)	(*Q*_*im*_,*Q*_1_)
1	318.9	(0,0)
2	352.1	(1,0)
3	990.7	(2,0)
4	1204.3	(3,0)
5	1694.1	(4,0)
6	2099.5	(5,0)
7	2473.9	(0,1)
8	2543.4	(1,1)
9	2601.6	(6,0)
10	3047.2	(7,0)
11	3155.5	(2,1)
12	3421.5	(3,1)
13	3544.1	(8,0)

### Asymmetric Double Wells

An interesting question is how the method and results vary when the double well potential is asymmetric. The asymmetry can be of two types, one where the PES has wells of equal depth but where differences in the ZPE (caused typically by isotopic substitution) create the asymmetry, and ones where the wells are of unequal depth. We will first present examples of the *Q*_*im*_ method for cases in which there are two wells with different electronic energies before returning to the former case.

Many theoretical investigations of tunneling in asymmetric wells have been presented; among them are refs 27,33–37 . The question here is not how to solve the quantum mechanics but rather how well the *Q*_*im*_-method works. Unfortunately, there are few experimental examples for asymmetric double wells, especially for molecules that are possible to study with high-precision PESs. In addition, as we will see, although the asymmetry separates the levels on the two sides of the barrier, the contribution to that separation due to the tunneling is decreased and thus often harder to detect experimentally. Medel et al. have studied hydrogen tunneling in alpha-fenchol, a large molecule that has nonequivalent minima of accidental near degeneracy.^38^ In the ground vibrational level, the tunneling splittings are 16 and 7 cm^–1^ for the torsional states. These are not exceedingly small, but the molecule is nearly symmetric. Farrell et al. have studied tunneling splitting in the HF-DF/DF-HF asymmetric double wells of the ground and excited states using high-resolution near-IR spectroscopy.^39^ The splittings are typically a few hundredths of a wavenumber.

As a demonstration of how the *Q*_*im*_ method works on asymmetric double wells, we performed calculations on *Q*_*im*_-paths modeled on that for the protonated oxalate anion. In the first two examples, the potential was taken as *V*(*Q*_*im*_) = *V*_oxalate_(*Q*_*im*_) + α*Q*_*im*_, where α is a positive number that lowers the left well and raises the right one. The barrier, measured from the average of the two minima, remains the same as that for the oxalate. Panels (a) and (b) in Figure 7 show energy levels and probability densities for α values of 0.3 and 3.48 cm^–1^, respectively. In the second two examples, the potential was the same, except that the barrier was adjusted to be 585 cm^–1^. Representative examples for this barrier and for α values of 0.1 and 1.0 cm^–1^ are shown in panels (c) and (d) of Figure 7, respectively. The notations in the panels give the value of α, of Δ*E*_0_/2, equal to half the energy difference between the two minima, and of Δ*ij*, the absolute energy difference between levels *i* and *j*. All energy values are in cm^–1^. It appears that, in principle, there is no complication in using the *Q*_*im*_ method for asymmetric barriers and that useful information can be obtained. For example, from the probability densities in panels (c) and (d), one can see that for the lowest pair of levels the only case where the H atom is tunneling between the two wells is when they are barely displaced in (c) (α = 0.1); if they are substantially displaced as in (d) (α = 1.0), there is almost no tunneling. Panel b) shows the interesting case when the energy of *v* = 0 of the right well is nearly degenerate with *v* = 1 of the left well. The probability densities show spreading over both wells. As one scans the “detuning” between the two levels (by changing α), one finds that the “resonance”, where the probability spans both wells, is quite narrow, on the order of a few cm^–1^, as shown in Figure 8. The solid green line shows the DVR solution with tunneling as a function of half the difference between the PES minima for the two wells (bottom abscissa). The dashed blue line gives the probability of being in the original well, while the solid blue line gives the probability of being in the other well. At resonance, all of the level splitting is due to the tunneling, and there is an equal probability of being in the left or right well. The dashed green lines in Figure 8 show what the level splitting would be without tunneling as a function of *d*, the separation between the two zero-order levels. Diagonalization of a Hamiltonian interaction matrix with diagonal elements ±*d* and off-diagonal elements of *ℏ*Ω has the solution $\Delta =2\sqrt{d^{2}+(\hbar \Omega {)}^{2}}$ , where the Δ is the observed splitting, ±*d* are the zero-order energy levels, and (*ℏ*Ω) is the contribution from the tunneling.

**Figure 7:**
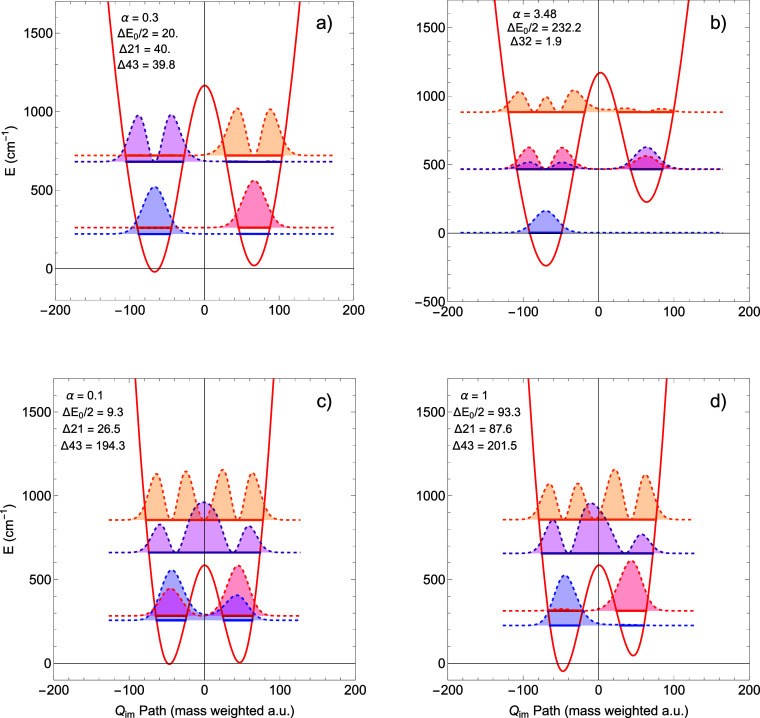
Energy levels and probability densities for asymmetric potentials for different barrier heights and degrees of asymmetry (see text for details).

**Figure 8:**
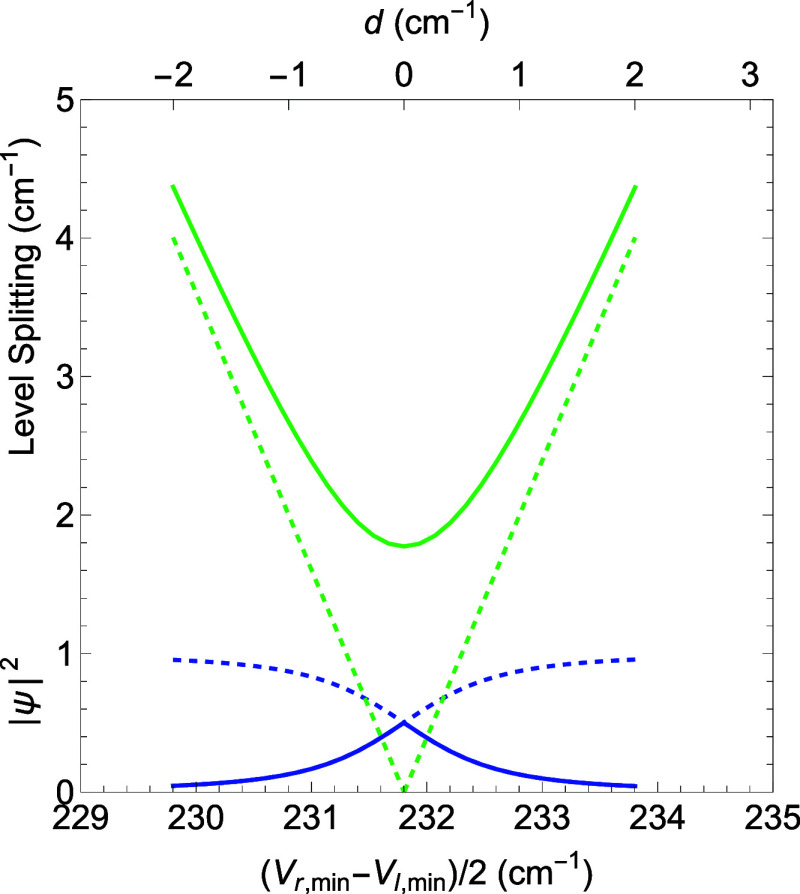
Scan of the difference between the potential energy minima of the right and left wells showing (solid green) the absolute half splitting between the PES minima level of the right and left wells. (see Figure 7c). The parameter *d* gives half the detuning of the levels from resonance. The solid blue curve gives the probability of being in the original well, while the dashed blue curve gives the probability of being in the other well. The dashed green lines show the contribution of the absolute level splitting due only to the asymmetry.

We now return to an example of the case where the electronic energy of the two wells is the same but where the asymmetry is caused by a difference in zero-point energies. This is the situation for a version of tropolone in which one of the oxygen atoms involved in the hydrogen transfer is ^16^O and the other is ^18^O (see Table 1). The *Q*_*im*_ method was used on this example by following the procedure of normal-mode analysis at the transition state, optimization of the *Q*_*im*_ -path, and use of DVR to calculation the energy levels, but all done now with the isotopic variant. For the asymmetric variant we obtained 4.27 cm^–1^, while for symmetric tropolone we obtained 1.66 cm^–1^. By examining the two wells separately using DVR, we found that the zero-order states are separated by 3.73 cm^–1^, with *d* equal to half this value. Given the value of Δ = 4.27 cm^–1^, and recalling that that $\Delta =2\sqrt{d^{2}+(\hbar \Omega {)}^{2}}$ , we determine that (*ℏ*Ω) = 1.23 cm^–1^. These values, while not in precise agreement with the RPI results,^30^ are qualitatively correct.

## Discussion

As seen in Table 1, nearly all of the *Q*_*im*_ results are in quite good agreement with those obtained by other “benchmark” methods. This is also the case for the challenging hydronium with the large amplitude umbrella motion, provided the vibrational angular momentum terms are added to the 1d *Q*_*im*_ Hamiltonian, as reported in ref 14. We note that the RPI and DMC methods used in the comparisons require substantially more effort than the 1d *Q*_*im*_ one. The calculations here do make use of full-dimensional PESs; these are needed for DMC and RPI calculations. But as we demonstrated, it is possible to use the *Q*_*im*_ method by calculating a minimum energy path without a PES using an electronic structure package. In these cases, one can then simply project this path onto the ${\vec{q}}_{im}$ direction calculated from the structure of the transition state and in this way avoid the necessity of determining a full-dimensional PES. This projection gives the relaxed *Q*_*im*_-path between the transition state and the minimum, but it does not provide the repulsive walls of the double well, which are needed to get accurate DVR calculations of the ZPE and energy level spacings. An approximate method for obtaining the repulsive part of the potential has been presented and discussed above.

The multidimensional *Q*_*im*_ method, demonstrated here for protonated oxalate and also previously,^14^ is a straightforward and effective method to determine the splittings of vibrational levels in addition to those of the imaginary frequency mode. Estimations of these have been made using a *Q*_*im*_ model in which the normal modes of a minimum are projected onto the ${\vec{q}}_{im}$ direction to determine the turning points on *V*(*Q*_*im*_) to calculate the change in tunneling for excitation.^6^ Nonetheless, this is still an area in need of further research.

We included the results from RPI calculations here as “benchmarks”, even though they are not full quantum ones. Instanton theory is a semiclassical method that dates to the late 1970s.^40^ Its application to realistic tunneling splittings was advanced by Mil’nikov and Nakamura.^41,42^ The theory does not assume a path, as is done in the *Q*_*im*_-method. Rather the goal of instanton theory is to locate optimum least-action path. In general this requires extensive knowledge of the full-dimensional potential surface.^43,44^ RPI^24,25^ and variations of it^27^ have provided a computationally efficient method to a search in high dimensional space of the potential. This is still a computationally expensive undertaking, certainly compared to the 1d *Q*_*im*_-path method. Suggestions for making this search efficient have been proposed.^45,46^ We do wish to stress that RPI is a major numerical advance in doing instanton calculations. Recent perturbation theory corrections to it have shown increased accuracy for the malonaldehyde splitting compared to true “benchmark” results.^47^

It is worth noting that the ground state tunneling splitting can be obtained from the exact low temperature partition function. This can be obtained using quantum path integral molecular dynamics.^48,49^ An interesting study of this approach, DMC and RPI for malonaldehyde points out the strength and weakness of this method, which has statistical uncertainties of the order of those from a DMC calculation.^50^

Finally, we provide some comments with respect to strengths and weaknesses of the 1d *Q*_*im*_ approach. The former include the simplicity and good quantitative accuracy for the range of splittings examined here. The biggest weakness in our view is the fixed choice of reaction path. As noted already, for acetylacetone the methyl rotors do not relax along the path. This is traceable to the limitations of the Watson Hamiltonian, which cannot describe internal rotors. However, the “fix” described above does provide a reasonable and expedient way to alter the *V*(*Q*_*im*_) potential. A second issue, is “corner cutting” paths cannot be described. These paths are known to be important in the deep tunneling region, with the formic acid dimer as a prime example. Using an accurate full-dimensional potential, two of us reported a ground state tunneling splitting of 0.44 cm^–1^ using the 1d-*Q*_*im*_ approach.^51^ As noted above, the RPI splitting using this potential is 0.014 cm^–1^ and is in very good agreement with experiment.^26^ As noted in that paper the instanton path is corner-cutting, i.e., does not pass through the saddle point. So while the absolute error in the 1d-*Q_im_* is small, it is clear that such a small tunneling splitting is beyond the expected accuracy of the simple 1d-*Q*_*im*_ approach. So, based on the present results and these for formic acid dimer, it appears that the simple approach cannot be expected to provide good quantitative accuracy for H-atom transfer tunneling splittings less than about 1 cm^–1^.

## Summary and Conclusions

We reviewed and extended the 1d *Q*_*im*_-path method to obtain tunneling splittings for symmetric and asymmetric double well potentials. Tests of the method for H-atom transfer in malonaldehyde, acetylacetone, tropolone, and protonated oxalate were given as well as a review of applications to umbrella motion tunneling in ammonia and hydronium. The method was shown to be accurate to within several wavenumbers. The extension to asymmetric double wells described asymmetry due to asymmetric isotopic substitution as well as intrinsic potential asymmetry. The former was tested for a specific isotopologue of tropolone, where the calculated splitting approximately doubles, in good agreement with experiment.

The 1d *Q*_*im*_-path method does not describe corner-cutting, which becomes increasingly important for tunneling splittings much less than 1 cm^–1^. For this reason, we do not expect the method to provide quantitative accuracy for such small splittings. This was exemplified in previous work for the formic acid dimer.^51^

## References

[1] Fukui K., Kato S., Fujimoto H. (1975). Constituent analysis of the potential
gradient along a reaction coordinate. Method and an application to
methane + tritium reaction. J. Am. Chem. Soc..

[2] Marcus R. A. (1966). On the
Analytical Mechanics of Chemical Reactions. Quantum Mechanics of Linear
Collisions. J. Chem. Phys..

[3] Truhlar D. G., Kuppermann A. (1972). Exact and
Approximate Quantum Mechanical Reaction Probabilities
and Rate Constants for the Collinear H + H2 Reaction. J. Chem. Phys..

[4] Fernández-Ramos A., Miller J. A., Klippenstein S. J., Truhlar D. G. (2006). Modeling the Kinetics
of Bimolecular Reactions. Chem. Rev..

[5] Miller W. H., Handy N. C., Adams J. E. (1980). Reaction
path Hamiltonian for polyatomic
molecules. J. Chem. Phys..

[6] Wang Y., Bowman J. M. (2013). Mode-specific tunneling
using the Qim path: Theory
and an application to full-dimensional malonaldehyde. J. Chem. Phys..

[7] Wang Y., Bowman J. M. (2008). One-dimensional
Tunneling Calculations in the Imaginary-frequency,
Rectilinear Saddle-point Normal Mode. J. Chem.
Phys..

[8] Watson J. K. G. (1968). Simplification
of the Molecular Vibration-Rotation Hamiltonian. Mol. Phys..

[9] Bowman J. M., Carter S., Huang X. (2003). MULTIMODE: a Code to Calculate Rovibrational
Energies of Polyatomic Molecules. Int. Rev.
Phys. Chem..

[10] Carter S., Bowman J. M., Handy N. C. (1998). Extensions and Tests of “Multimode”:
A Code to Obtain Accurate Vibration/Rotation Energies of Many-Mode
Molecules. Theor. Chem. Acc..

[11] Léonard C., Handy N. C., Carter S., Bowman J. M. (2002). The vibrational
levels of ammonia. Spectrochim. Acta, Part A.

[12] Huang X., Carter S., Bowman J. (2003). Ab initio
potential energy surface
and rovibrational energies of H_3_O^+^ and its isotopomers. J. Chem. Phys..

[13] Wang Y., Braams B., Bowman J. M., Carter S., Tew D. P. (2008). Full-dimensional
Quantum Calculations of Ground-state Tunneling Splitting of Malonaldehyde
Using an Accurate ab initio Potential Energy Surface. J. Chem. Phys..

[14] Kamarchik E., Wang Y., Bowman J. (2009). Reduced-Dimensional
Quantum Approach
to Tunneling Splittings Using Saddle-Point Normal Coordinates. J. Phys. Chem. A.

[15] Hammer T., Manthe U. (2011). Intramolecular proton transfer in malonaldehyde: Accurate
multilayer multi-configurational time-dependent Hartree calculations. J. Chem. Phys..

[16] Light, J. C. ; Carrington, T. Discrete Variable Representations and their Utilization. In Adv. Chem. Phys.; Wiley: 2003; Vol. 114, pp 263–310.

[17] Colbert D. T., Miller W. H. (1992). Large-amplitude Dynamics in Vinyl Radical: The Role
of Quantum Tunneling as an Isomerization Mechanism. J. Chem. Phys..

[18] Houston P. L., Conte R., Qu C., Bowman J. M. (2020). Permutationally
Invariant Polynomial Potential Energy Surfaces for Tropolone and H
and D atom Tunneling Dynamics. J. Chem. Phys..

[19] Tanaka K., Honjo H., Tanaka T., Kohguchi H., Ohshima Y., Endo Y. (1999). Determination of the proton tunneling splitting of tropolone in the
ground state by microwave spectroscopy. J. Chem.
Phys..

[20] Keske J. C., Lin W., Pringle W. C., Novick S. E., Blake T. A., Plusquellic D. F. (2006). High-resolution
studies of tropolone in the S_0_ and S_1_ electronic
states: Isotope driven dynamics in the zero-point energy levels. J. Chem. Phys..

[21] Firth D. W., Beyer K., Dvorak M. A., Reeve S. W., Grushow A., Leopold K. R. (1991). Tunable far-infrared
spectroscopy of malonaldehyde. J. Chem. Phys..

[22] Baba T., Tanaka T., Morino I., Yamada K. M. T., Tanaka K. (1999). Detection
of the tunneling-rotation transitions of malonaldehyde in the submillimeter-wave
region. J. Chem. Phys..

[23] Qu C., Houston P. L., Bowman J. M. (2026). Does the
diffuse OH-stretch band
in the IR spectrum of protonated oxalate exhibit quantum chaos?. J. Chem. Phys..

[24] Richardson J. O., Althorpe S. C. (2011). Ring-polymer Instanton
Method for Calculating Tunneling
Splittings. J. Chem. Phys..

[25] Richardson J. O. (2018). Perspective:
Ring-Polymer Instanton Theory. J. Chem. Phys..

[26] Richardson J. O. (2017). Full- and
reduced-dimensionality instanton calculations of the tunnelling splitting
in the formic acid dimer. Phys. Chem. Chem.
Phys..

[27] Eraković M., Cvitaš M. T. (2022). Vibrational
Tunneling Spectra of Molecules with Asymmetric
Wells: A Combined Vibrational Configuration Interaction and Instanton
Approach. J. Chem. Theory Comput..

[28] Qu C., Conte R., Houston P. L., Bowman J. M. (2021). Full-dimensional
Potential Energy Surface for Acetylacetone and Tunneling Splittings. Phys. Chem. Chem. Phys..

[29] Qu C., Houston P. L., Conte R., Nandi A., Bowman J. M. (2021). Breaking
the Coupled Cluster Barrier for Machine-Learned Potentials of Large
Molecules: The Case of 15-Atom Acetylacetone. J. Phys. Chem. Lett..

[30] Nandi A., Laude G., Khire S. S., Gurav N. D., Qu C., Conte R., Yu Q., Li S., Houston P. L., Gadre S. R. (2023). Ring-Polymer Instanton Tunneling Splittings
of Tropolone and Isotopomers using a *Δ*-Machine
Learned CCSD­(T) Potential: Theory and Experiment Shake Hands. J. Am. Chem. Soc..

[31] Andreichev V., Käser S., Bocanegra E. L., Salik M., Johnson M. A., Meuwly M. (2025). Dynamics of protonated oxalate from machine-learned
simulations and experiment: infrared signatures, proton transfer dynamics
and tunneling splittings. Phys. Chem. Chem.
Phys..

[32] Qu, C. ; Houston, P. L. ; Yu, Q. ; Nandi, A. ; Bowman, J. M. ; Andreichev, V. ; Käser, S. ; Meuwly, M. State-of-the-Art Analysis of the Complex IR Spectrum of Protonated Oxalate from Full Dimensional Machine Learned Potentials, in preparation.

[33] Miller W. H. (1979). Periodic
orbit description of tunneling in symmetric and asymmetric double-well
potentials. J. Phys. Chem..

[34] Cribb P. H., Nordholm S., Hush N. S. (1979). A density
matrix approach to double
well transfer: effects of asymmetry on the tunneling rate. Chem. Phys..

[35] Yamada K. M. T., Ross S. C. (2006). Isomerisation: Don’t forget
the possibility
of enhanced tunnelling! A simple two-state model for the on-resonance
and near-resonance behaviour of enhanced tunnelling. J. Mol. Struct..

[36] Jahr E., Laude G., Richardson J. O. (2020). Instanton
theory of tunneling in
molecules with asymmetric isotopic substitutions. J. Chem. Phys..

[37] Yang Y.-T., Chen S.-M., Luo H.-G. (2025). On the
origin of the hidden symmetry
in the asymmetric quantum Rabi model. J. Chem.
Phys..

[38] Medel R., Springborn J. R., Crittenden D. L., Suhm M. A. (2022). Hydrogen Delocalization
in an Asymmetric Biomolecule: The Curious Case of Alpha-Fenchol. Molecules.

[39] Farrell J., John T., Suhm M. A., Nesbitt D. J. (1996). Breaking symmetry
with hydrogen bonds: Vibrational predissociation and isomerization
dynamics in HF–DF and DF–HF isotopomers. J. Chem. Phys..

[40] Miller W.
H. (1975). Semiclassical
limit of quantum mechanical transition state theory for nonseparable
systems. J. Chem. Phys..

[41] Mil’nikov G. V., Nakamura H. (2001). Practical implementation
of the instanton theory for
the ground-state tunneling splitting. J. Chem.
Phys..

[42] Mil’nikov G. V., Kühn O., Nakamura H. (2005). Ground-State and Vibrationally Assisted
Tunneling in the Formic Acid Dimer. J. Chem.
Phys..

[43] Rommel J. B., Goumans T. P. M., Kästner J. (2011). Locating Instantons in Many Degrees
of Freedom. J. Chem. Theory Comput..

[44] Zaverkin, V. ; Kästner, J. Tunnelling in Molecules: Nuclear Quantum Effects from Bio to Physical Chemistry; The Royal Society of Chemistry: 2020.

[45] Cooper A. M., Hallmen P. P., Kästner J. (2018). Potential
energy surface interpolation
with neural networks for instanton rate calculations. J. Chem. Phys..

[46] Laude G., Calderini D., Tew D. P., Richardson J. O. (2018). Ab initio
instanton rate theory made efficient using Gaussian process regression. Farad. Discuss..

[47] Lawrence J. E., Dušek J., Richardson J. O. (2023). Perturbatively corrected ring-polymer
instanton theory for accurate tunneling splittings. J. Chem. Phys..

[48] Vaillant C. L., Wales D. J., Althorpe S. C. (2019). Tunneling
Splittings in Water Clusters
from Path Integral Molecular Dynamics. J. Phys.
Chem. Lett..

[49] Zhu Y.-C., Yang S., Zeng J.-X., Fang W., Jiang L., Zhang D. H., Li X.-Z. (2023). Accurate calculation of tunneling
splittings in water clusters using path-integral based methods. J. Chem. Phys..

[50] Baumann J., Trenins G., Richardson J. O. (2025). The exact
tunnelling splitting of
malonaldehyde from symmetrized path-integral molecular dynamics. Mol. Phys..

[51] Qu C., Bowman J. M. (2016). An ab initio potential
energy surface for the formic
acid dimer: zero-point energy, selected anharmonic fundamental energies,
and ground-state tunneling splitting calculated in relaxed 1–4-mode
subspaces. Phys. Chem. Chem. Phys..

